# Past, Present and Future: The Relationship Between Circular RNA and Immunity

**DOI:** 10.3389/fimmu.2022.894707

**Published:** 2022-05-25

**Authors:** Junjie Gu, Chongying Su, Fei Huang, Yuwei Zhao, Jing Li

**Affiliations:** ^1^ State Key Laboratory of Oral Diseases, National Clinical Research Center for Oral Diseases, Chinese Academy of Medical Sciences Research Unit of Oral Carcinogenesis and Management, West China Hospital of Stomatology, Sichuan University, Chengdu, China; ^2^ Chengdu Blood Center, Blood Research Laboratory, Chengdu, China

**Keywords:** circRNAs, immunity, immune-related diseases, autoimmune diseases, tumor, infectious diseases

## Abstract

The immune system has evolved since the birth of humans. However, immune-related diseases have not yet been overcome due to the lack of expected indicators and targeting specificity of current medical technology, subjecting patients to very uncomfortable physical and mental experiences and high medical costs. Therefore, the requirements for treatments with higher specificity and indicative ability are raised. Fortunately, the discovery of and continuous research investigating circular RNAs (circRNAs) represent a promising method among numerous methods. Although circRNAs wear regarded as metabolic wastes when discovered, as a type of noncoding RNA (ncRNA) with a ring structure and wide distribution range in the human body, circRNAs shine brilliantly in medical research by virtue of their special nature and structure-determined functions, such as high stability, wide distribution, high detection sensitivity, acceptable reproducibility and individual differences. Based on research investigating the role of circRNAs in immunity, we systematically discuss the hotspots of the roles of circRNAs in immune-related diseases, including expression profile analyses, potential biomarker research, ncRNA axis/network construction, impacts on phenotypes, therapeutic target seeking, maintenance of nucleic acid stability and protein binding research. In addition, we summarize the current situation of and problems associated with circRNAs in immune research, highlight the applications and prospects of circRNAs in the treatment of immune-related diseases, and provide new insight into future directions and new strategies for laboratory research and clinical applications.

## 1 Introduction

**Figure 1:**
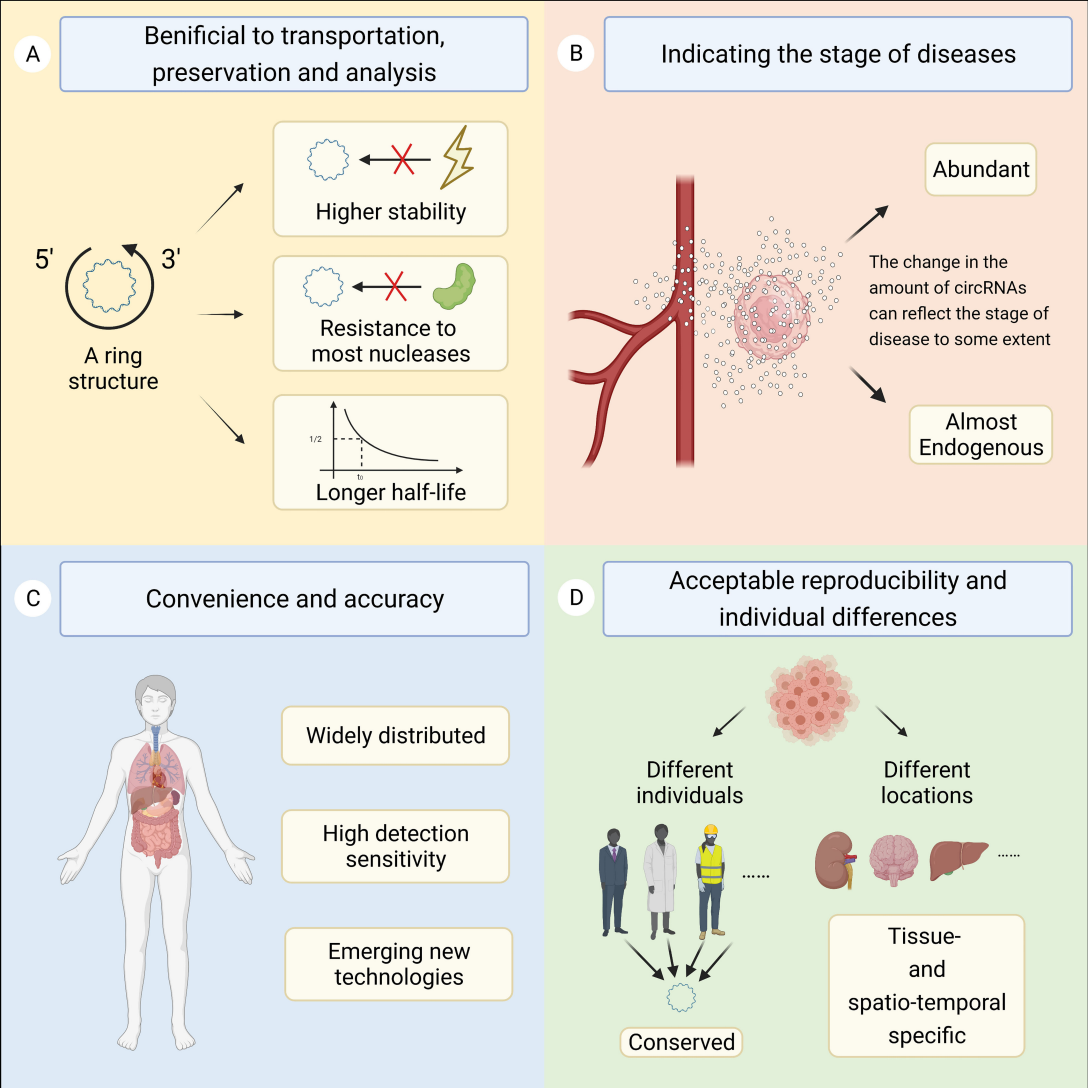
This figure shows the four main characteristics of circRNAs. **(A)** The ring structure is beneficial for the transportation, preservation, and analysis of samples. **(B)** The change in the amount of circRNAs can reflect the stage of disease to some extent. **(C)** CircRNAs are widely distributed and have high detection sensitivity. **(D)** CircRNAs are conserved, tissue- and spatiotemporal specific, resulting in acceptable reproducibility and individual differences.

CircRNAs are molecules belonging to the noncoding RNA family that form ring-like structures with covalent bonds without 5’ caps and 3’ poly (A) tails (1). CircRNAs were first found in pathogens but were regarded as meaningless or even incorrect expression products for decades. In recent years, researchers have begun to realize the importance of circRNAs with the rapid development of specific biochemical and computational methods, such as high-throughput sequencing technology and microarray techniques (2, 3). CircRNAs are generally stable and thought to have unique structural conformations that differ from their linear RNA homology (4). As confirmed, circRNAs are principally formed *via* the junction of a downstream 3’ site with an upstream 5’ site, generated *via* back-splicing or exon skipping of premRNAs in general (5, 6). Over years of research, circRNAs have been found to feature four main characteristics (**Figure 1**). First, circRNAs are connected from end to end to form ring structures, enhancing their stability and resistance to most ribonucleases. Studies have shown that the half-life of circRNAs is longer than that of corresponding linear transcripts, which is beneficial for the transportation, preservation, and analysis of samples. Second, circRNAs are conserved, tissue- and spatiotemporal specific, resulting in acceptable reproducibility and individual differences (7). Third, circRNAs are abundant and almost endogenous (8, 9). The expression level of circRNAs changes accordingly under physiological or pathological conditions. Therefore, the change in the amount of circRNAs can reflect the stage of disease to some extent. Fourth, circRNAs are widely distributed and have high detection sensitivity (8). Currently, circRNAs are commonly divided into the following four categories according to their constituent sequences: exonic circRNAs (ecircRNAs), circular intronic RNAs (ciRNAs), exon–intron circRNAs (EIciRNAs) and tRNA intronic circular RNAs (tricRNAs). However, the circRNAs currently found are mainly derived from exons (5, 9).

Since the advent of empirical immunology, research investigating immunity has lasted for a long time. The immune system plays a dual role in the fight against diseases. On the one hand, the body’s immune barrier acts as a defense against intruders, and a functioning immune system can quickly detect and address abnormal conditions in the body. On the other hand, a dysregulated response of the human immune system may lead to the deterioration of the disease or the emergence of autoimmune diseases. With the development of research, scientists have noticed that circRNAs are of vital importance in human immunity and have potential clinical significance in the diagnosis and treatment of autoimmune diseases, tumor immunity, infectious diseases and other immune-related diseases (9–12).

This review expounds upon the progress and existing problems in this field and provides potential development directions for the future to improve the environment for clinical treatment.

## 2 Biological Functions of circRNAs

According to current studies, circRNAs participate in different physiological processes of human diseases and perform a wide range of functions as miRNA sponges, transcription templates, special protein binding sites and regulators of host genes. Many current studies have focused on circRNAs in the cytoplasm, and some of those circRNA were reported to act as competing endogenous RNAs (ceRNAs) and usually function as sponges for miRNAs, thereby regulating miRNAs targeting gene expression (13, 14). A typical example is CDR1as, namely, ciRS-7, which contains more than 70 miRNA response elements (MREs) for miR-7 and can combine with miR-7 to downregulate its miRNA actions (15–17). In addition, circRNAs can use their specific regions to interact with proteins (18), function as protein scaffolds (19) and recruit specific proteins to certain locations in cells (20); thus, circRNAs with internal ribosome entry sites (IRESs) and infinite open reading frames (ORFs) can be translated under specific circumstances (21, 22). Moreover, circRNAs correlated with RNA polymerase II (Pol II) in human cells, localizing in the nucleus, could modulate the expression of their host genes (23, 24). However, unfortunately, research concerning the function of circRNAs only focuses on a small fraction of circRNAs that have been found, thereby requiring more specificity. Therefore, there is still more development space for other types of circRNAs that are less studied, rending the future of this field full of uncertainty and promises.

## 3 Roles of circRNAs in Immune-Related Diseases

**Figure 2:**
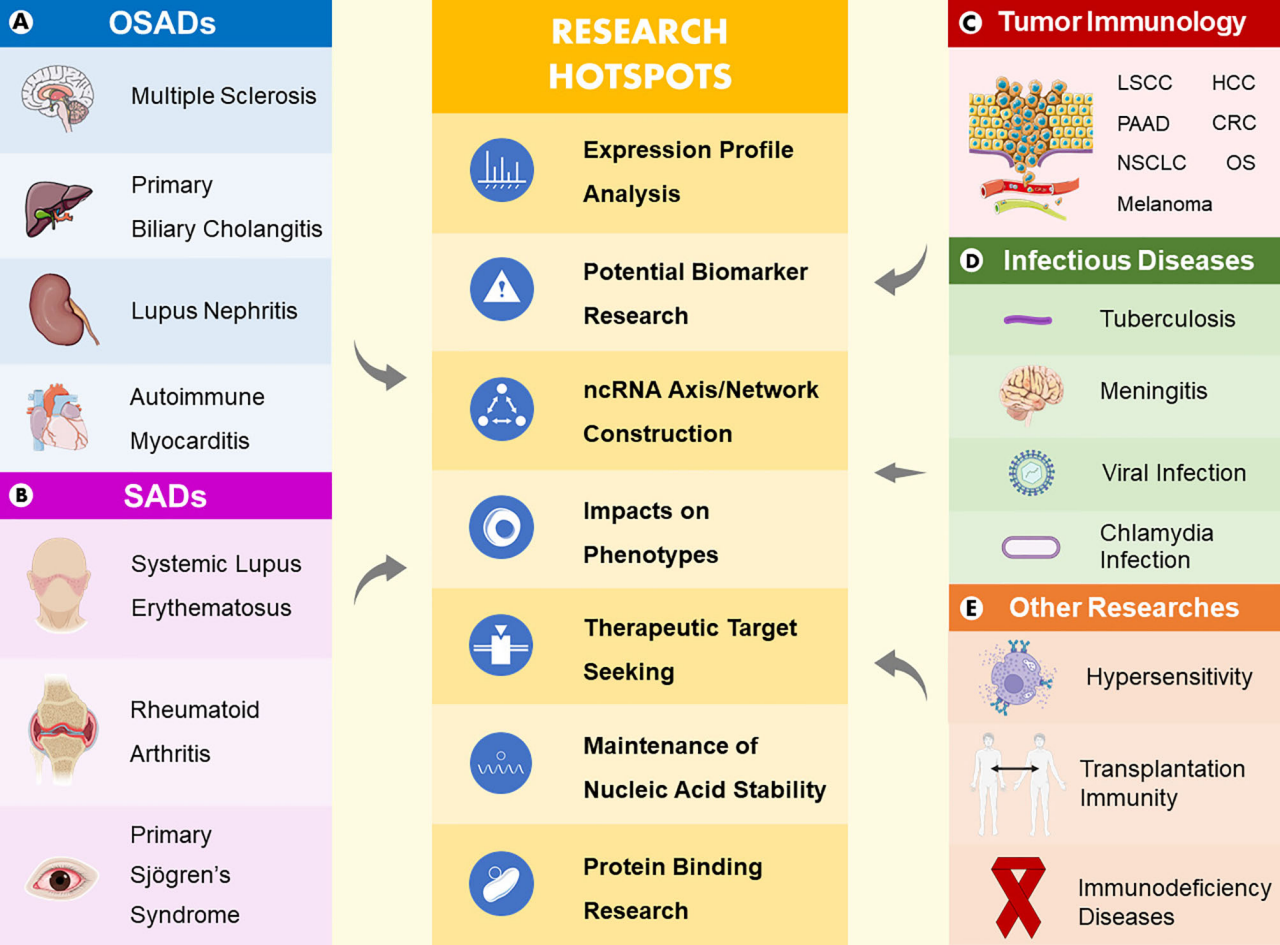
Main areas and research hotspots of the roles of circRNAs in immune-related diseases. Five main areas consist of organ-specific autoimmune diseases **(A)**, systemic autoimmune diseases **(B)**, tumor immunology **(C)**, infectious diseases **(D)**, and other studies **(E)**. In summary, research hotspots of the roles of circRNAs in immune-related diseases include expression profile analyses, potential biomarker research, ncRNA axis/network construction, impacts on phenotypes, therapeutic target seeking, maintenance of nucleic acid stability and protein binding research.

Many studies have been performed to uncover the mechanism of immunity with the purpose to solve the problems of immunological diseases. With continuous research development, increasing evidence has emerged showing that circRNAs are able to intervene in the biological processes of assorted immune-related diseases by acting as miRNA sponges, protein interactors, mRNA stability maintainers, potential biomarkers and therapeutic targets *via* diverse axes and intricate signaling pathways (**Table 1**). In this section (**Figure 2**), we describe new experimental progress in circRNAs that participate in organ-specific autoimmune diseases (OSADs), systemic autoimmune diseases (SADs), tumor immunology, and infectious diseases and summarize the roles of circRNAs in other studies.

**Table 1:** Roles of circRNAs in four main immune-related diseases.

Diseases	circRNAs	Functions	Related molecules	Related pathways	Ref
Organ-specific autoimmune diseases					
Multiple sclerosis	circ_0005402	Potential biomarker	——	——	(25)
	circ_0035560				
	hsa_circ_0106803	Potential biomarker	——	——	(26)
Primary biliary cholangitis	hsa_circ_402458	Potential biomarker	——	——	(27)
Lupus nephritis	circ_002453	Potential biomarker	——	——	(28)
Autoimmune myocarditis	circSnx5	miRNA sponge	miR-544, SOCS1, PU.1	JAK/STAT signaling pathway, MAPK signaling pathway	(29)
Systemic autoimmune diseases					
Systemic lupus erythematosus	hsa_circ_0045272	miRNA sponge	IL-2, hsa-miR-6127	Apoptosis signaling pathway	(30)
	hsa_circ_0000479	Potential biomarker	——	——	(31)
	hsa_circ_0044235	Potential biomarker	——	——	(32)
	hsa_circ_0068367				
	circPTPN22	Potential activity indicator	——	——	(33)
	hsa_circ_407176	Potential biomarker	——	——	(34)
	hsa_circ_001308				
Rheumatoid arthritis	hsa_circ_0001200	Potential biomarker	——	——	(35)
	hsa_circ_0001566				
	hsa_circ_0003972				
	hsa_circ_0008360				
	hsa_circ_0000396	Potential biomarker	——	——	(36)
	hsa_circ_0130438				
	hsa_circ_0088036	miRNA sponge	miR-140-3p, SIRT1	AMPK signaling pathway, mTOR signaling pathway	(37)
Primary Sjögren's syndrome	hsa_circ_001264	Potential biomarker	——	——	(38)
	hsa_circ_104121				
	hsa_circ_045355				
Tumor immunology					
Laryngeal squamous cell carcinoma	hsa_circ_001569	miRNA sponge	CD274, IL-10, FOXP3	Th17 cell differentiation	(39)
	hsa_circ_001859				
Pancreatic adenocarcinoma	circUBAP2	miRNA sponge	CXCR4, ZEB1	Wnt signaling pathway, MAPK signaling pathway	(40)
Melanoma	circ_0020710	miRNA sponge	miR-370-3p, CXCL12	mTOR signaling pathway	(41)
Non-small cell lung cancer	circMET	miRNA sponge	miR-145-5p, CXCL3	TNF signaling pathway	(42)
	circFGFR1	miRNA sponge	miR-381-3p, CXCR4	HIF-1 signaling pathway, Wnt signaling pathway	(43)
Hepatocellular carcinoma	circUHRF1	miRNA sponge, potential biomarker	miR-449c-5p, IFN-γ, TNF-α, TIM-3	TNF signaling pathway, MAPK signaling pathway	(44)
Colorectal cancer	circSPARC	miRNA sponge, protein binder, potential biomarker and therapeutic target	miR-485-3p, JAK2, STAT3, FUS	JAK/STAT signaling pathway	(45)
Infectious diseases					
Pulmonary tuberculosis	hsa_circ_14623	miRNA sponge, potential biomarker	——	Endocytosis pathways in cancer, MAPK signaling pathway, HTLV-1 infection, and ubiquitin- mediated proteolysis signaling pathway	(46)
	hsa_circ_09585				
	hsa_circ_005538				
	hsa_circ_09993				
	hsa_circ_00074				
	hsa_circ_13478				
	hsa_circ_0005836	potential biomarker and therapeutic target	——	——	(47)
Active tuberculosis	hsa_circ_001937	potential biomarker	——	——	(48)
Bacterial meningitis	hg38_circ_0002276	miRNA sponge	hsa-miR-548o-3p	——	(49)
	hg38_circ_0031043		hsa-miR-548o-3p		
	hg38_circ_ 0027134		hsa-miR- 148a-3p		
	hg38_circ_ 0032477		hsa-miR- 148a-3p		
	hg38_circ_0008980		hsa-miR-660-5p		
	hg38_circ_0001582		hsa-miR-194-5p		
	hg38_circ_0017427		hsa-miR-107		
Viral infection	circRNAs	interacting with proteins	K63, RIG-I, IRF3, NF90/110	Innate immunity	(50, 51)
COVID-19	Ppp1r10	miRNA sponge	mmu-miR-124-3p, Ddx58, Gm26917	Antiviral mechanism by IFN-stimulated genes	(52)
	C330019G07RiK				
Microbial infection	circRasGEF1B	maintaining the stability of the mature mRNA	ICAM-1, LPS, NF-κB	LPS pathway, NF-κB pathway, cell cycle, macrophage polarization	(53)
Chlamydia infection	hsa_circ_001226	miRNA sponge	——	Endocytosis, MAPK and PI3P-Akt signaling pathway	(54)
	hsa_circ_007046				
	hsa_circ_400027				

### 3.1 CircRNAs in Organ-Specific Autoimmune Diseases

In OSADs, autoantigens are a specific component of an organ, and the pathological damage and dysfunction of tissue are limited to the organ targeted by antibodies or lymphocytes. Multiple sclerosis (MS) is an autoimmune disease that demyelinates the white matter of the central nervous system. Although the specific pathogenesis remains unclear, circRNAs have been found to participate in the progression of MS (55). Cardamone et al. indicated that the abnormal metabolism of circRNAs is a potential characteristic of MS (26). Moreover, Iparraguirre et al. found two downregulated circRNAs and considered them potential biomarkers of MS (25). Among ncRNAs, miRNAs and lncRNAs are currently popular issues in MS, while circRNAs are relatively less studied; thus, more research is needed to supplement the regulatory network of ncRNAs in MS (56).

In addition to MS, circRNAs play an important role in many other OSDAs. For example, circRNAs showed promise as candidate biomarkers of primary biliary cholangitis (27), and plasma circRNA_002453 could be used as a novel biomarker of lupus nephritis (28). Researchers also found that circSnx5 control the immunogenicity of dendritic cells as a miRNA sponge, thereby alleviating experimental autoimmune myocarditis (29). Although the participation of circRNAs has further increased the complexity of the mechanism of OSAD, current research is still very simple, and it is difficult to promote the understanding of this type of disease and the therapeutic application of circRNAs.

### 3.2 CircRNAs in Systemic Autoimmune Diseases

Systemic autoimmune disease is a type of systemic multiple organ damage caused by the extensive deposition of antigen and antibody complexes on the vascular wall, and systemic lupus erythematosus (SLE) is a common disease. The exact cause of SLE is still unclear, but circRNAs have recently been regarded as vital molecules in SLE. Li et al. compared the different circRNA profiles in T cells from healthy and ailing patients and then revealed the biofunction of hsa_circ_0045272 (30). Currently, many circRNAs have been identified as biomarkers of SLE (31–34). Recently, Zhang et al. retrieved the GEO database and obtained a regulatory network, providing novel insight into the role of circRNAs in SLE (57).

Another common type of systemic autoimmune disease is rheumatoid arthritis (RA), whose pathogenesis has not been fully elucidated thus far. Currently, in the research field of circRNAs in RA, research mainly focuses on expression profile analyses, biomarker research and the proliferation and migration of fibroblast-like synovial cells. Wen et al. constructed a network of differentially expressed circRNAs and miRNAs and eventually revealed the expression profile of peripheral blood mononuclear cells (PBMCs) in patients with RA (35). Yang et al. used RNA sequencing technology to uncover the circRNA expression profiles of PBMCs in experimental and control groups and found that circRNAs are novel diagnostic markers of RA (36). Regarding the proliferation and migration of fibroblast-like synovial cells, Zhong et al. found that hsa_circ_0088036 promoted the proliferation and migration of fibroblast-like synovial cells *via* the miR-140-3p/SIRT1 axis in RA (37). In addition to SLE and RA, circRNAs are of great concern in some SADs. For example, Su et al. reported that hsa_circ_001264 might be a biomarker of primary Sjögren’s syndrome (pSS) (38). Although circRNAs are closely connected to SADs such as SLE, RA and pSS, research concerning SADs, such as scleroderma, dermatomyositis and polymyositis, currently mainly focuses on miRNAs. Consequently, there is substantial untapped potential in research investigating the mechanisms of circRNAs and SADs.

### 3.3 CircRNAs in Tumor Immunology

Currently, the study of tumor immunology focuses on the body’s immune response to tumors, the mechanism of tumor immune escape tumor immune diagnosis and immune prevention. Over years of research, scientists have discovered that circRNAs are very important molecules in various tumors, playing a variety of immunological functions. The hottest research area is the competing endogenous RNA (ceRNA) network and its regulatory molecules. Sun et al. constructed ceRNA networks based on 133 laryngeal squamous cell carcinoma (LSCC) patients and found that hsa_circ_001569 and hsa_circ_001859 might regulate the expression of CD274, IL-10 and FOXP3, thus intervening in the immune escape of LSCC (39). In another study on ceRNA networks in pancreatic adenocarcinoma (PAAD), Zhao et al. reported that CXCR4 and ZEB1 were regulated by the circUBAP2-mediated ceRNA network, inhibiting antigen presentation and promoting tumor immune escape (40). Among studies investigating ceRNA networks, research focusing on the circRNA/miRNA/mRNA axis is especially plentiful. The effects on cell phenotypes are mainly reflected in the ability to drive tumor immune escape and promote proliferation and metastasis *via* different axes (41, 42). In addition, studies related to anti-PD-1 therapy are included in these reports (43, 44).

With the deepening of understanding, researchers have discovered the potential of circRNAs as therapeutic targets and biomarkers with abilities to assist with diagnosis and prognosis and their function in the immune regulation of exosomes. Wang et al. showed that circSPARC might conceivably act as a possible biomarker for diagnosis and prognosis and a target for therapy in colorectal cancer (CRC) (45). In another study concerning hepatocellular carcinoma (HCC), scientists reported that the upregulated level of plasma exosomal circUHRF1 decreased NK-cell tumor infiltration, curbing the function of NK cells (44).

Generally, from the laboratory to the clinical level, current research focusing on circRNAs in tumor immunology is proceeding in an orderly manner. Therefore, the application of circRNAs in the future may have a very positive impact on many aspects of tumor immunotherapy, such as diagnosis, treatment, and prognosis.

### 3.4 CircRNAs in Infectious Diseases

Infectious diseases refer to diseases in which bacteria, viruses, fungi, parasites, and other infectious agents, invade, grow, and reproduce in the body, causing damage to the normal metabolic functions of the tissue structure. Thus far, circRNAs have been found to be used as biomarkers of many infectious diseases in most cases, while emergent corroboration indicates that a small number of circRNAs are verified to directly impact the regulatory network of infectious diseases (58). By regulating the NF-κB pathway, the potential miRNA targets of hsa_circ_001937 exert effectiveness in antibacterial immune responses in patients with tuberculosis (46, 48). In a bioinformatics analysis experiment, Zhuang et al. found that hsa_circ_0005836 could be a novel biomarker for diagnosis and prognosis and a target for therapy of active pulmonary tuberculosis (47). In a similar experiment, Yang et al. performed a circRNA transcription analysis of primary human brain microvascular endothelial cells infected with meningeal Escherichia coli and preliminarily constructed a potential regulatory network that enhanced our understanding of the mechanisms of bacterial meningitis (49). Additionally, in Marinov et al.’s study focusing on an LPS-inducible circRNA called circRasGEF1B, the authors assumed that inducible RasGEF1B circular RNA may play an essential role in protecting cells against microbial infection by preserving the constancy of the mature mRNA of ICAM-1 in LPS-activated cells (53), providing a new idea for antimicrobial infection therapies. CircRNAs are of vital importance to the infection of viruses because the abnormal expression of circRNAs may promote or suppress the infection progress of viruses, and vice versa. Studies have shown that when infected with viruses, circRNAs are rapidly degraded by RNase L, releasing PKR (dsRNA-activated protein kinase) linked to circRNAs and participating in innate cellular immune responses (35, 59). Notably, circRNAs initiate innate immunity by combining with K63, which links ubiquitin chains, and RIG-I (retinoic acid-inducible gene I). Exogenous circRNAs without m6A modification can attach to K63 and RIG-I. This complex can promote the polymerization and activation of RIG-I, affect the aggregation of downstream mitochondrial antiviral signals, guide the dimerization and activation of interaction regulating factor 3 (IRF3), and finally induce the expression of autoimmune-related pathway genes (50, 60, 61). The antiviral dsRNA-binding protein NF90/110 can stabilize the secondary structure of intronic RNA, thereby promoting the biogenesis of circRNAs. NF90/110 can also act as global regulators of circRNA biogenesis by reducing their nuclear levels during viral infection (51, 62).

Since the end of 2019, the world has experienced several rounds of outbreaks caused by variants of severe acute respiratory syndrome coronavirus 2 (SARS-CoV-2) and improper anti-epidemic measures. Omicron, a newly discovered SARS-COV-2 variant with high transmission, is causing unease and uncertainty. Therefore, whether focusing on the present or the future, it is particularly important to develop new treatment technologies on the basis of existing epidemic prevention measures such as drug development and vaccination (63). CircRNAs, molecules closely associated with viral infection, is one option. A differential host circRNA expression profile analysis in human lung epithelial cells infected with SARS-CoV-2 was completed (64), and two circRNA profile analyses revealed abundant and diverse information regarding the identification and characterization of the circRNAs encoded by SARS-CoV-1, SARS-CoV-2 and MERS-CoV (65, 66), facilitating future studies concerning on COVID-19 infection and pathogenesis. Arora et al. identified a circRNA/lncRNA-miRNA–mRNA ceRNA network involving two circRNAs in SARS-CoV-2-infected cells, enhancing the current understanding of the mechanisms associated with coronavirus disease 2019 (COVID-19) (52). Specific segments of the SARS-CoV-2 5’-untranslated region can be expeditiously accessed by particular antisense circRNAs, resulting in bringing an approximately 90% cutback in virus proliferation in cell culture with a minimal duration of 2 days, which is attractive and promising (67). Briefly, relevant research focused on expression profile analyses, ceRNA construction and therapeutic target seeking. Although circRNAs are in the initial stage in the prevention and treatment of novel coronavirus, with the development of cross-discipline and the emergence of more advanced technology, it is believed that there will be opportunities for circRNAs to display their clinical talents in the future.

In addition to bacteria and viruses, circRNAs function in many other infectious diseases, such as chlamydia infection (54). In addition to modulating the human body, circRNAs can play a regulatory role in other organisms during infectious diseases, such as parasite infection. Broadbent et al. identified developmentally regulated lncRNAs and circRNAs by strand-specific RNA sequencing in Plasmodium falciparum malaria (68), but currently, there is no clinical significance.

Overall, current research concerning circRNAs in infectious diseases mostly focuses on viral and bacterial infections, but in addition to research as biomarkers, these results are still a long way from clinical application.

### 3.5 CircRNAs in Other Immunological Research

Thus far, we mentioned that circRNAs are of great significance in autoimmune diseases, tumors, and bacterial and viral infections. In addition, expanding the perspective to the whole area, circRNAs perform effectively in hypersensitivity, immunodeficiency diseases and transplantation immunity, namely, the pathological changes caused by immune defense function. However, because the contents and categories of current related studies are relatively similar, there are only a few examples, which are no longer explained in detail here. For instance, circHIPK3 was proven to modulate the proliferation of airway smooth muscle cells by the miR-326/STIM1 axis in asthma (69), a group of ample circRNAs and ceRNA networks were found to likely contribute to acquired immune deficiency syndrome (AIDS) (70), and a two-circular RNA signature of donors was thought to be a biomarker of early allograft dysfunction after liver transplantation (71). In addition to the above diseases, circRNAs play vital roles in a variety of immunological diseases and immune cells. Under stimulation by different pathological factors, the way that circRNAs are involved in the activation of macrophages is a large subject (53, 72–74). In addition, a variety of circRNAs have been identified to influence various immune cells, such as intestinal immune cells (75), lung immune cells poisoned by Nd2O3 (76), CD4+ T cells in asthma (77) and immune cells in periodontitis (78). In addition to these effects on different immune cells, there are also some studies focusing on innovative technologies. Recently, Wesselhoeft et al. showed that unmodified exogenous circRNA can bypass cellular RNA sensors, thereby avoiding immune responses in RIG-1- and Toll-like receptor (TLR)-competent cells and mice, suggesting that RNA circularization reduces immunogenicity and can prolong the translation time *in vivo (* 61).

Studies concerning circRNAs in immune-related diseases are miscellaneous, but the core functions and mechanisms are constant. The discovery of circRNAs has further deepened researchers’ understanding of the intricate immune regulatory network. Generally, the immune system has three major functions, namely, immune defense, immune surveillance and immune homeostasis, and circRNAs realize immune-related mechanisms as follows: 1) during immune defense functions, circRNAs can assist the body in removing pathogenic microorganisms and other antigens in various ways; however, hypersensitivity or immune deficiency occurs when the immune response is too high or too low; 2) when immune surveillance operates regularly, circRNAs can help the body remove mutant cells and virus-infected cells through various pathways; if this function is abnormal, it could lead to tumor occurrence and persistent virus infection; and 3) when immune homeostasis occurs naturally, circRNAs can aid the body in removing damaged or senescent cells in various ways, but imbalance could lead to autoimmune diseases. Therefore, the balance between the immune system and circRNAs plays a key role in whether the body is in a healthy or pathological state.

### 3.6 Regulatory Mechanisms of circRNAs in Immune-Related Diseases

**Figure 3:**
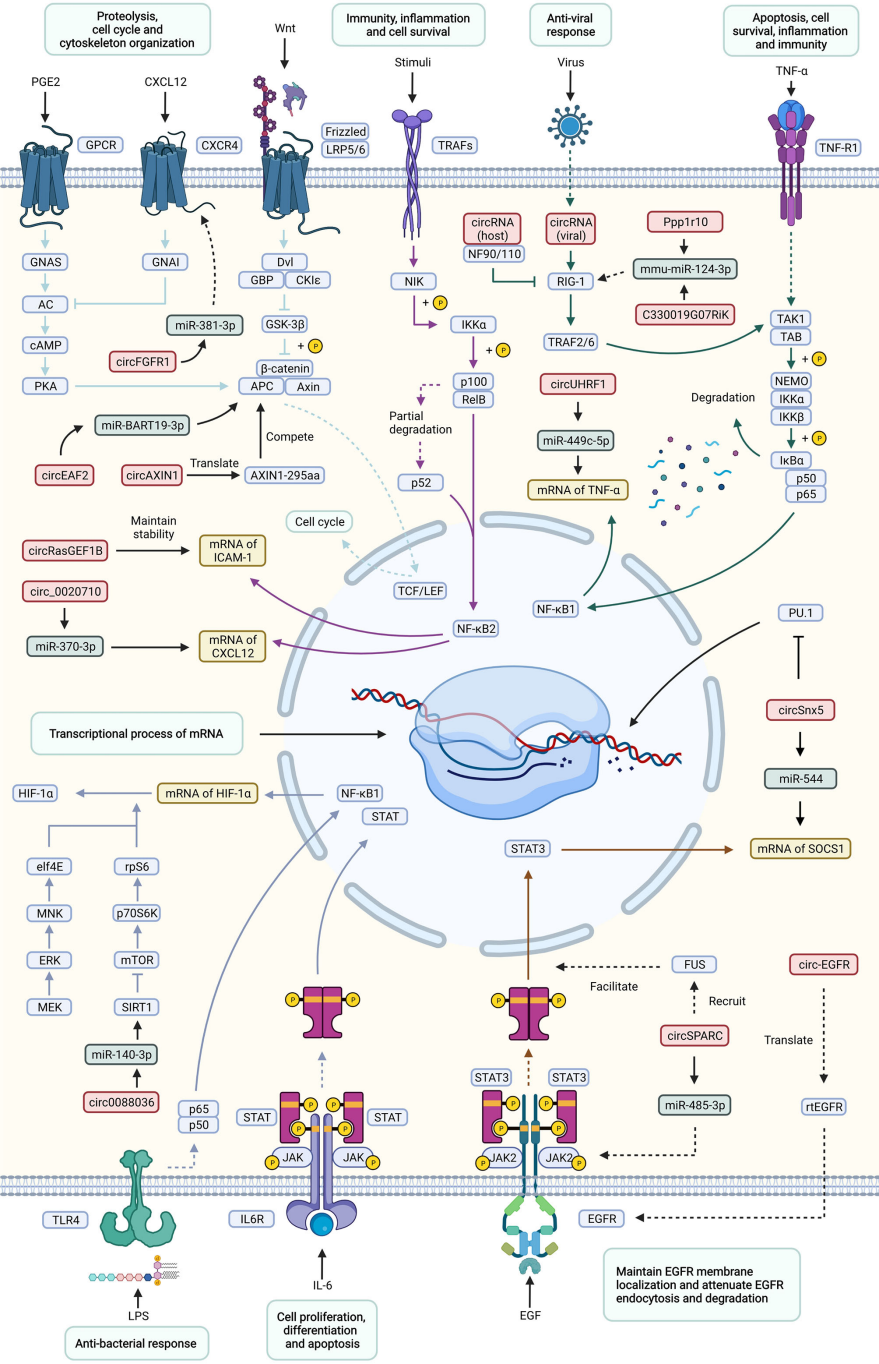
Important signaling pathways of circRNAs involved in the regulation of immune-related diseases. This figure shows how circRNAs influence immune-related diseases *via* a variety of signaling pathways, including the Wnt, TNF, NF-κB, JAK-STAT, mTOR, antiviral and antibacterial pathways. and the corresponding responses. Effects and processes are shown in light green rectangles, circRNAs are shown in red rectangles, miRNAs are shown in dark green rectangles, mRNAs are shown in yellow rectangles, and proteins are shown in blue rectangles. Different signaling pathways are distinguished by arrows and inhibitors of different colors. Solid lines represent direct interactions between molecules, while dotted lines represent indirect interactions.

The regulatory mechanism of circRNAs in immune-related diseases can be summarized into the following two aspects: the regulatory effects of circRNAs on immune-related signaling pathways (**Figure 3**), such as the MAPK signaling pathway, endocytosis signaling pathway, JAK-STAT signaling pathway, mTOR signaling pathway, and Wnt signaling pathway, and the regulatory effects of circRNAs on immune cells, such as the regulation of macrophages, etc.

### 3.7 Important Signaling Pathways of circRNAs Involved in the Regulation of Immune-Related Diseases

#### 3.7.1 MAPK Signaling Pathway

The MAPK signaling pathway is a signal transduction system important for eukaryotic cells to mediate extracellular signals in the intracellular response. This pathway transduces extracellular signals in the form of a triple kinase cascade, namely, MAP kinase kinase kinase (MKKK), MAP kinase kinase (MKK) and MAP kinase (MAPK), which regulates a variety of physiological processes, such as cell growth, differentiation, apoptosis and death. There are four main branches of the MAPK pathway as follows: extracellular-signal regulated protein kinase (ERK), c-Jun N-terminal kinase (JNK), p38 mitogen-activated protein kinase (p38 MAPK) and ERK5. Among them, JNK and p38 have similar functions, which are related to inflammation, apoptosis and cell growth; ERK is mainly responsible for cell growth and differentiation, and its upstream signals are the famous Ras and Raf proteins (79, 80). According to current studies, circRNAs mainly play roles as miRNA sponges in the MAPK signaling pathway in immune-related research. For example, Chen et al. found that circSnx5 acted as a sponge of miR-544 to upregulate suppressor of cytokine signaling 1 (SOCS1) (81). Zhang et al. revealed the circUHRF1/miR-449c-5p/TIM-3 axis in HCC (44). Zhao et al. constructed a ceRNA network consisting of 4 DEcircRNAs, 3 DEmiRNAs and 149 DEmRNAs in PAAD (40), which also showed the sponge function of circRNAs. Among the regulated proteins, SOCS1 (82–84), TIM-3 (85–87), ZEB1 (88–90), etc., serve as important regulators in the MAPK signaling pathway.

#### 3.7.2 Endocytosis Signaling Pathway

Endocytosis is the process of transporting extracellular substances into cells through the deformed movement of the plasma membrane. Endocytosis can be divided into phagocytosis, pinocytosis and receptor-mediated endocytosis according to the size and mechanism. According to clathrin dependence, endocytosis can be divided into clathrin-dependent endocytosis (CDE) and clathrin-independent endocytosis (CIE). In terms of trends, the mechanism of the relationship between signal transduction and endocytosis has received increasing attention in studies investigating of the occurrence and development of many diseases. Endocytosis has been proven to be closely related to lipid metabolism, intracellular iron homeostasis, metabolism, immunity and other functions (91–93). On the basis of existing research, circRNAs mainly participate in the endocytosis pathway as miRNA sponges in immune-related research. Abnormally expressed circRNAs were identified in pulmonary tuberculosis (46) and chlamydia infection (54), and all were predicted to be miRNA sponges. Through bioinformatics analyses, these circRNAs were found to be related to the endocytosis signaling pathway. A special study focused on the protein translation function of circRNAs, and verified that circ-EGFR attenuates EGFR endocytosis and degradation (94).

#### 3.7.3 JAK-STAT Signaling Pathway

The JAK-STAT signaling pathway has been revealed to consist of the following four parts: extracellular signaling factors, tyrosine kinase-related receptors, tyrosine kinase called Janus kinase (JAK) that transmits signals, and transducer and activator of transcription (STAT) that exerts effects. When a variety of cytokines and growth factors bind receptors, JAK is activated, and then the activated JAK phosphorylates the receptor and itself. These phosphorylated sites become the binding sites of STAT with an SH2 structure, thus recruiting and phosphorylating STAT and allowing it to enter the nucleus in the form of a dimer to bind to target genes, regulating the transcription of downstream genes and modulating the process of cell proliferation, differentiation and apoptosis (95, 96). In light of research conducted thus far, circRNAs mainly act as miRNA sponges in the JAK-STAT signaling pathway in immune-related research. For instance, Wang et al. uncovered the circSPARC/miR-485-3p/JAK2 axis in CRC (45). In type 1 diabetes mellitus, Yang et al. identified the hsa_circ_0060450/miR-199a-5p/mRNAs axis, which suppressed the JAK-STAT signaling pathway triggered by IFN-I (97).

#### 3.7.4 mTOR Signaling Pathway

Mammalian target of rapamycin (mTOR) is an evolutionarily conserved serine/threonine protein kinase that can regulate a variety of cell functions by phosphorylating its downstream target protein. There are two key complexes in the mTOR signaling pathway called mTOR complex 1 (mTORC1, including mTOR, Raptor, mLST8, etc.) and mTOR complex 2 (mTORC2, including mTOR, Rictor, mLST8, etc.). mTORC1 is activated in the presence of lysosome levels, ER stress, sterols, hypoxia and energy stress to regulate several biological processes, including lipid metabolism, autophagy, protein synthesis and ribosomal biogenesis, while mTORC2 responds to growth factors and controls cytoskeletal organization, metabolism and cell survival (98–100). According to studies, circRNAs mainly exert an influence as miRNA sponges in the mTOR signaling pathway in immune-related research. For example, Zhong et al. revealed the circ0088036/miR-140-3p/silent information regulator 1 (SIRT1) axis in the promotion of RA (37). Wei et al. indicated the importance of the circ_0020710/miR-370-3p/CXCL12 axis in melanoma (41). Regarding the regulated proteins, SIRT1 (101–103), CXCL12 (104–106), etc., served as important regulators in the mTOR signaling pathway.

#### 3.7.5 Wnt Signaling Pathway

The Wnt signaling pathway is a complex regulatory network that has been verified to include at least the following three branches: the classical Wnt signaling pathway, namely, the Wnt/β-catenin signaling pathway, Wnt/planar cell polarity (PCP) pathway and Wnt/Ca2+ pathway activated by Wnt5a and Wnt11. Wnt mainly transmits signals through 7 transmembrane receptors of the Frizzled family and LRP5/6 coreceptors and plays a regulatory role in cells through key molecules such as CK1, Deshevelled, GSK3, APC, Axin, and β-Catenin (107–109). Currently, circRNAs mainly produce marked effects as miRNA sponges in the Wnt signaling pathway in immune-related research. For instance, Zhang et al. stated that the circFGFR1/miR-381-3p/CXCR4 axis promoted NSCLC progression and resistance to anti-programmed cell death 1 (PD-1)-based therapy (43). Zhao et al. proposed that circEAF2 counteracts Epstein–Barr virus-positive diffuse large B-cell lymphoma progression *via* the miR-BART19-3p/APC/β-catenin axis (110). Regarding the regulated proteins, CXCR4 (111–113), APC (114–116), etc., served as important regulators in the Wnt signaling pathway. Specifically, a study revealed that a novel protein AXIN1-295aa encoded by circAXIN1 activated the Wnt/β-catenin signaling pathway to promote gastric cancer progression (117).

In addition to the pathways highlighted above, circRNAs participate in the regulation of the TNF, AMPK, HIF-1 and NF-κB. signaling pathways, but generally, the mechanisms are similar; thus, circRNAs exert effects on immune function and immune-related diseases mainly by translating proteins and acting as miRNA sponges.

#### 3.7.6 Regulation of circRNAs in Immune Cells

CircRNAs have various regulatory functions and have been detected in different types of immune cells, such as macrophages, dendritic cells (DCs), natural killer cells (NK cells), CD4+ T cells and CD8+ T cells. By inhibiting or promoting the activation or exhaustion of these cells, circRNAs participate in the development of various diseases.

#### 3.7.7 Regulation of circRNAs in Macrophages

CircRNAs affect the activation of macrophages. For instance, mouse macrophages specifically express circ-RasGEF1B in the form of NF-κB after being stimulated by lipopolysaccharide (LPS), which can activate macrophages by positively regulating the expression of intercellular adhesion molecule-1 (ICAM-1) (53, 72). Zhang et al. found that circPPM1F participates in the activation of MI macrophages in diabetic patients (118), while another study showed that hsa_circ_0110102 inhibits macrophage activation *via* the miR-580-5p/PPARα/CCL2 pathway (119). In addition, SiO_2_ induces macrophage activation through the circHECTD1/HECTD1 pathway and circZC3H4 RNA and ZC3H4 protein in the process of pulmonary fibrosis (120, 121). In addition, circRNA HIPK3 and circUbe3a can activate macrophages, while the latter participates in the process of myocardial fibrosis (122, 123).

Furthermore, circRNAs can lead to the polarization of tumor-associated macrophages to M1 or M2 macrophages. One study showed that circN4 bp1 could act as a miR-138-5p sponge for the modulation of macrophage polarization through the regulation of the expression of EZH2 (a histone methyltransferase) (124). Moreover, circRNA Cdyl, circPrkcsh and circPPM1F were found to play a role in inducing M1 macrophage polarization (118, 125, 126). Many studies have highlighted the importance of circRNAs in the occurrence and development of tumors, and one effect is the mediation of the polarization of M2 macrophages. For example, tumor-derived extracellular circFARSA was discovered to mediate the polarization of M2 macrophages (127). Additionally, cyclic RNA PLCE1, circITGB6, circ_0001142 and hsa_circ_0074854 were also found to play such a role (128–131).

CircRNAs also play a role in regulating the macrophage-related inflammatory response; for example, hsa_circ_0005567 can promote M2 macrophage polarization *via* the mir-492/SOCS2 axis (132). Moreover, hsa_circ_0004287 inhibits macrophage-mediated inflammation in an N-methyladenosine-dependent manner in atopic dermatitis and psoriasis (133). Furthermore, circRNAs can also advance the inflammatory response. In gouty arthritis, circHIPK3 was found to be able to activate the macrophage inflammasome (134), as did hsa_circ_0087352, circ_1639 and circ_0001490 (135–137).Significantly, in Mycobacterium tuberculosis infection, circRNAs TRAPPC6B and hsa_circ_0045474 can induce autophagy in macrophages (138, 139). Other studies have found that the circRNA calcitonin gene-related peptide (CGRP) can induce macrophages to express IL-6 (140).

#### 3.7.8 The Regulation of circRNAs on Other Immune Cells

Current research investigating the correlation between circRNAs and immune cells mainly focuses on macrophages, and there are relatively few studies of other cells. Here, we briefly review the regulation of circRNAs in NK cells, DCs, CD4+ T cells and CD8+ T cells.

CircRNAs can promote NK-cell depletion and regulate cytotoxicity. A study found that hsa_circ_0048674 and cancer cell-derived exosome circUHRF1 can induce NK-cell dysfunction (44, 141). Hsa_circ_0007456 regulates NK-cell-mediated hepatocellular carcinoma cytotoxicity through the mir-6852-3p/ICAM-1 axis (131). Moreover, circARSP91 can enhance innate immune surveillance by strengthening the cytotoxicity of NK cells (142). In addition, circrHT1 knockout can aggravate the sensitivity of bladder cancer cells to NK cells, and another study showed that circ_0000977 knockout can enhance the killing effect of NK cells on pancreatic cancer cells through HIF1A and ADAM1 (143, 144). A GO analysis showed that circRNAs were involved in regulating DC differentiation and other biological functions (145). Chen et al. found that circSnx5 controls the immunogenicity of DCs through the miR-544/SOCS1 axis (29). Furthermore, Wang et al. discovered that the knockdown of circFSCN1 could affect the ability of DCs to activate T cells and enhance Treg generation (146). Another study showed that growth differentiation factor 15 induces tolerant DCs (Tol DCs) by inhibiting the circ_malat-1 and NF-κB signaling pathways and upregulating IDO (147).

Research investigating the connection between circRNAs and CD4+ T cells mainly concentrates on systemic lupus erythematosus (SLE) and asthma. Studies have shown that the DNA methylation of CD11a and CD70 in CD4 T cells form patients with SLE is associated with the downregulation of hsa_circ0012919 (148). In addition, the regulatory network among circHIPK3, LncGAS5 and miR-495 can promote Th2 differentiation in allergic rhinitis (149), and hsa_circ_0002594 and hsa_circ_0005519 can affect asthma by regulating CD4+ T cells (77, 150). Moreover, N-methyladenosine-modified circIGF2BP3 was found to inhibit CD8+ T-cell responses and promote tumor immune evasion (151), while exogenous circTRPS1 was proven to be related to CD8+ T-cell exhaustion (152). In addition, Chen et al. noted that the expression of circRNA100783 is affected by time- and CD28-related CD8(+) T-cell aging during antigen exposure (153). Clinically, cancer cell-derived exosomal circUSP7 was proven to induce CD8+ T cell dysfunction and anti-PD1 resistance by regulating the miR-934/SHP2 axis in NSCLC (154).

### 3.8 Applications and Prospects of circRNAs in the Treatment of Immune-Related Diseases

Immunotherapy refers to a treatment technique that artificially heightens or represses the immune function of the body to treat immune-related diseases in accordance with the low or hyperactive immune state of the body. Because of their unique structure and various functions, circRNAs have broad application prospects in the treatment of immune-related diseases.

At the current stage, most studies investigating the functions of circRNAs are still in the laboratory stage, and only a few theories have been developed for technical applications in clinical treatment, such as gene therapy. Tens of thousands of studies have proven circRNAs to be substantially considerable in the advancement of many immune-related diseases, suggesting the roles of circRNAs as therapeutic agents and targets (50, 62, 147, 155–157). To date, there are four main approaches to realizing gene therapy as follows: inducing or inhibiting the expression of the target circRNA upstream, chemically modifying key molecules, designing analogs of the target circRNA and designing downstream molecular analogs of the circRNA, i.e., miRNA.

In addition to gene therapy, with the discovery of the function of encoding proteins, circRNAs are speculated to have the potential to be novel drug delivery carriers. Wesselhoeft et al. produced a protein with high quality and stable expression in eukaryotic cells after the circularization of mRNA *in vitro* and indicated that RNA circularization can reduce immunogenicity and extend translational duration in vivo (61, 158, 159), providing insight into the treatment of immune-related diseases. In addition, circRNAs have the potential to function as appropriate biomarkers of immune-related diseases. In case of immune-related diseases, it is usually difficult for patients to determine whether they fell ill by the clinical symptoms in the early stage so that they will not go to the hospital until their symptoms worsen (9). Therefore, circRNAs can function as ideal biomarkers due owing to the four main characteristics mentioned above. Thus far, numerous circRNAs have been found in exosomes, and changes in the content of circRNAs in exosomes can reflect the process of diseases (159). However, the current problem that has blocked the application of circRNAs as biomarkers in the clinic is that with the continuous improvement of the circRNA database in immune-related diseases, the expression of the same circRNA in different diseases may have the same trend, which may interfere with the judgment. Therefore, a more refined database needs to be established.

Unparalleled strides have been made in cancer treatment with the use of immune checkpoint blockade (ICB), but ICB resistance hinders the efficacy of cancer immunotherapies (160). Based on existing research, regulating gene expression at the transcriptional level, acting as miRNA sponges, binding functional proteins and encoding proteins are the four major biological functions of circRNAs, and these functions can play a vital role in regulating immune diseases, such as immune escape, immune tolerance, and antitumor and anti-infection effects, either independently or in combination (7, 12, 161–167). Moreover, circRNAs can achieve cross-cellular regulation *via* exosomes. Recent studies have certified the potential role of exosomes in tumor immunity and resistance to ICB (160). For instance, Lu et al. suggested that immuno-repression and anti-PD1 resistance were caused by exosomal circTMEM181 by increasing the expression of CD39, and suppressing the ATP-adenosine signaling pathway by targeting CD39 on macrophages could rescue anti-PD1 therapy resistance in HCC (168). Therefore, *via* exosomes, circRNAs may yield unusually brilliant clinical results in ICB.

Considering that specially designed antisense circRNAs can effectively access the SARS-CoV-2 5’-untranslated region and inhibit the proliferation of most viruses for a time, circRNAs also an option for the clinical treatment of COVID-19, which is a major achievement that uses of the unique structure of circRNAs and artificial assistance for modification, showing many advantages. The best advantage is that the antisense sequence of circRNAs is better than the corresponding linear configuration and modified antisense oligonucleotides, and antisense circRNAs have strong activity against point mutations in the target sequence. This approach manifests the function of circRNAs as nucleic acid binders, starting novel applications for designing circRNAs and hopeful therapeutic strategies for COVID-19 (67). Fortunately, Qu et al. designed a circular RNA vaccine encoding the receptor domain (RBD) of the spike protein of SARS-CoV-2 for the very virus and its mutants and found that the circRNARBD-Delta vaccine designed for the SARS-CoV-2 Delta mutant was a candidate vaccine for COVID-19 with broad-spectrum protection in rhesus monkeys. A series of comparative evaluations showed that compared with mRNA vaccines, circRNA vaccines have higher stability and a higher proportion of neutralizing antibodies, which can effectively reduce the potential side effects of vaccine-associated respiratory diseases (VAERD) (169).

Despite numerous studies, research focusing on circRNAs is still limited, and many problems remain to be solved. Although the structure of circRNAs can help attenuate off-target effects, this problem cannot be avoided. Moreover, a specific circRNA may have different functions in different cells and may cause uncontrollable side effects. In addition, if an exogenous circRNA is synthesized without protein-binding partners, it may be recognized by RIG-I as a virus-derived circRNA and thus induce innate immunity (7–9, 62).

### 3.9 Discussion and Perspectives

CircRNAs perform the functions of sponging miRNAs, binding specific proteins and regulating gene transcription, and some can even encode proteins. Meanwhile, circRNAs are widely distributed in cells, the internal environment and exosomes, coupled with stable ring structures; thus, they have application potential in the diagnosis, treatment and prognosis of immune-related diseases. However, current research investigating related diseases mainly focuses on tumor immunity, bacterial and viral infections, and some autoimmune diseases, while relatively uncommon diseases are rarely studied. The hotspots of the roles of circRNAs in immune-related diseases include expression profile analyses, potential biomarker research, ncRNA axis/network construction, impacts on phenotypes, therapeutic target seeking, maintenance of nucleic acid stability and protein binding research. In addition, the study of the mechanism of circRNAs in immune regulation only occupies the tip of the iceberg in immunology. Currently, few studies on the regulation of circRNAs in the establishment of the immune system and the regulation of the immune system in the normal physiological state. A representative study showed that the structure and decomposition of circRNAs modulate PKR activation in innate immunity (4). At present, this field also faces some unsolved problems, such as off-target effects and unpredictable side effects. Therefore, continuing to supplement the regulatory network of circRNAs, attempting to explore new mechanisms, and developing new functions will be crucial for the entire field in the future, and the birth of new technologies will further contribute to the complete unveiling of the roles of circRNAs in immunity and immune-related diseases.

## Author Contributions

JG, CS and FH wrote the manuscript, designed the figures, collected the related references and edited the manuscript, and JL and ZY provided guidance and revised this manuscript. All authors approved the final manuscript.

## Funding

This work was supported by grants from the National Natural Science Foundation of China (81872211 and 82072999), the Sichuan Science and Technology Program (2020YJ0102), the Innovation Research Project of Sichuan University (2022SCUH0029), and the CAMS Innovation Fund for Medical Sciences (2020-I2M-C&T-A-023).

## Conflict of Interest

The authors declare that the research was conducted in the absence of any commercial or financial relationships that could be construed as a potential conflict of interest.

## Publisher’s Note

All claims expressed in this article are solely those of the authors and do not necessarily represent those of their affiliated organizations, or those of the publisher, the editors and the reviewers. Any product that may be evaluated in this article, or claim that may be made by its manufacturer, is not guaranteed or endorsed by the publisher.
